# Lipopolysaccharide exacerbates infarct size and results in worsened post-stroke behavioral outcomes

**DOI:** 10.1186/s12993-015-0077-5

**Published:** 2015-10-13

**Authors:** Danielle N. Doll, Elizabeth B. Engler-Chiurazzi, Sara E. Lewis, Heng Hu, Ashley E. Kerr, Xuefang Ren, James W. Simpkins

**Affiliations:** Department of Physiology and Pharmacology, Center for Basic and Translational Stroke Research, West Virginia University Health Science Center, 1 Medical Center Drive, Morgantown, WV 26506-9229 USA

**Keywords:** Stroke, LPS, Infection, Behavior, Post-stroke outcome

## Abstract

**Background:**

A third of ischemic stroke cases have no traditional underlying causes such as hypertension, diabetes, atherosclerosis, obesity, or age. Moreover, thirty to forty percent of strokes occur during or acutely after an active infection and the incidence of stroke increases during flu season. We and others have shown that the combination of a minor bacterial infection mimic, 100 μg/kg of lipopolysaccharide (LPS) prior to a minor stroke—30 min transient middle cerebral artery occlusion (tMCAO)—exacerbates infarct volume in a mouse model. Thus, experimental and epidemiological data strongly suggest that infection and/or inflammation play a role in stroke occurrence and severity. However, to date, long-term outcomes of stroke during an active infection has not been studied.

**Methods:**

3–4 month old C57Bl6/J mice were treated with saline or LPS 30 min prior to a 30 min tMCAO or sham surgery. A behavioral battery was administered to assess health status/sickness behavior, neurological deficits, motor, cognitive, and affective behaviors.

**Results:**

We show for the first time that exposure to a low dose of LPS prior to a mild stroke significantly worsens neurological deficits and sickness scores. Motor, cognitive, and affective behaviors were assessed post-stroke and while stroke significantly affected motor behavior on rotarod, LPS did not increase the motor deficits. We did not observe any effects of stroke or LPS on cognitive and affective behaviors.

**Conclusions:**

Our observations of the association between infection, stroke, and worse sickness and neurological outcomes identify (1) a clinical need to aggressively treat infections in people with risk factors for stroke and (2) the need to understand the mechanism(s) of the association between infections and stroke.

**Electronic supplementary material:**

The online version of this article (doi:10.1186/s12993-015-0077-5) contains supplementary material, which is available to authorized users.

## Background

Stroke is the 5th leading cause of death in the United States and the leading cause of disability [1]. Numerous risk factors such as diabetes, hypertension, and atherosclerosis have been recognized to increase susceptibility to stroke; however, the epidemiology of ischemic stroke is not sufficiently explained by the prevalence of these cerebrovascular risk factors [2]. In at least a third of ischemic stroke cases, the patients lack traditional risk factors and have no apparent cause. The presence of systemic infection could be an important mediating risk factor. Indeed, thirty to forty percent of strokes occur during or acutely after an infection [2] suggest an association between systemic infection and stroke. Although infection has been implicated in increasing susceptibility to stroke, few studies have examined how an infection prior to stroke affects outcome. McColl et al. [3] and Doll et al. [4] found that administering lipopolysaccharide (LPS) 30 min prior to transient middle cerebral artery occlusion (tMCAO) resulted in an increased infarct volume. We have shown that a mechanism by which lipopolysaccharide (LPS) exacerbates stroke damage involves an increase in blood brain barrier (BBB) damage through mitochondrial dysfunction [4] and others have suggested a cytokine-dependent mechanism [3]. Together, these findings suggest an association between systemic infection and stroke.

While it is clear that bacterial infection prior to stroke can affect acute stroke damage [3, 4], to our knowledge no studies have examined the long-term effects of a bacterial infection mimic on behavioral outcomes. We hypothesized that LPS administered prior to stroke would exacerbate the motor, cognitive, and affective behavioral deficits post-stroke. To test our hypothesis, we used an animal model of mild infection and mild stroke developed by McColl et al. [3], then assessed sickness behavior, neurological deficits, locomotor, cognitive, and affective behaviors to determine if a bacterial infection can exacerbate detrimental post-stroke functional outcomes.

## Methods

### Subjects

C57/BL6 J male mice (3–4 months old, 25–30 g, Jackson Laboratories) were used for all studies. All procedures were conducted according to criteria approved by the Institutional Animal Care and Use Committees at the West Virginia University.

### Randomization and blinding of the animal experiments

To assign groups (Vehicle Sham, LPS Sham, Vehicle Stroke, LPS Stroke), we numbered the animals and applied a simple randomization by using excel-generated random numbers. To avoid biases, we also assured that sham and stroke surgeries along with the different treatments (LPS or vehicle) were performed on the same day. The experimenters were blinded to the pre-treatments and data analysis.

### Drug administration

LPS (Escherichia coli 055:B5, 100 μg/kg, Sigma) dissolved in saline (B. Braun Medical Inc. Irvine, California) was administered via an intraperitoneal injection 30 min prior to tMCAO or sham surgery. An equal volume of saline was administered to vehicle treated mice.

### Ischemic model and sham surgery

We performed focal cerebral ischemia for 30 min by occlusion of the right middle cerebral artery with a 6.0 monofilament suture (Doccol, Sharon, Massachusetts). All surgical anesthesia was induced with 4–5 % isoflurane until the animal showed no response to a toe pinch, and was maintained with 1–2 % isoflurane via face-mask in O_2_-enriched air. We used laser Doppler flowmetry (Moor instruments, United Kingdom) to detect regional cerebral blood flow and confirm a successful occlusion (>70 % decrease in flow). Rectal body temperature was maintained at 37 ± 0.5 °C during surgery. One cohort of mice was euthanized at 48 h post-stroke to assess infarct area, and a separate cohort was euthanized at the end of the behavioral battery.

### Analysis of brain infarct area: cohort 1

In one cohort, a total of 28 mice (N = 7/group) were euthanized with isoflurane 48 h post-ischemia. We removed the brains and cut 2 mm coronal sections with a mouse brain matrix. We stained the sections with 2 % 2,3,5-triphenyltetrazolium chloride (TTC, Sigma, Saint Louis, Missouri) in phosphate buffer solution (PBS) at 37 °C for 30 min then fixed the tissue in 10 % formalin phosphate buffer for digital photograph. We analyzed the digitized image of each brain section using computerized image analysis software (ImageJ, NIH) in a double-blinded manner. Additional data from this cohort of animals was published in Doll et al. [4].

### Behavioral test battery: cohort 2

**Fig. 1:**
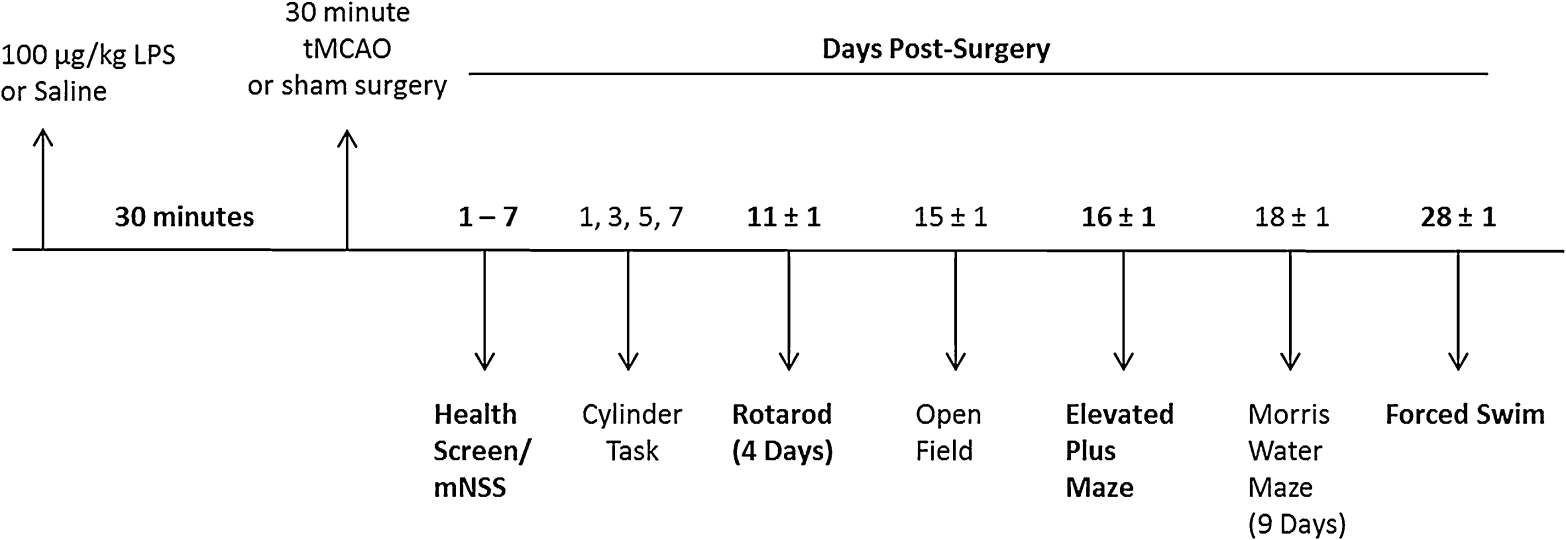
Timeline schematic of treatment, surgery and behavioral testing

In another cohort, a total of 40 mice (Vehicle Sham = 10; LPS Sham = 10; Vehicle Stroke = 10; LPS Stroke = 10) underwent behavioral testing. Two animals were excluded from the LPS stroke group; one due to post-mortem evidence of cerebral hemorrhage, and a second due to a surgery complication. The animal excluded due to a surgery complication was replaced resulting in an n = 9 for the LPS stroke group. The animals were treated and assessed in two cohorts with n = 5 in each group for each cohort. We selected the following behavioral tests to assess a range of affective, locomotor, and cognitive abilities and to identify and control for potential locomotor, visual, or motivational alterations effected by stroke and bacterial infection prior to stroke. Figure 1 depicts the time-course of behavioral testing. All animals underwent the entire test battery with the exception of Morris water maze. The first cohort showed no significant effects or trends toward a treatment or surgery effect on this test; thus, we did not subject the second cohort of animals to Morris water maze testing. However, the timing post-stroke of the other behavioral assessments were maintained constant across cohorts. All testing was completed during the light cycle (0800–1500) by the same experimenter, who was blind to treatment group status. Central air conditioning fans provided white noise in the testing room. Between each animal, the behavior apparatus was cleaned of debris with Virkon and, when appropriate (Open Field, Elevated Plus Maze), 50 % ethanol.

### Health and sickness behavior screen

Overall health and sickness behavior was assesses using an objective 20 point screen developed in our laboratory. The health screen was administered daily by the same experimenter who was blind to treatment group status. The screen encompasses seven physical domains designed to provide insight into the global physical health of an animal. The screen was designed to be rapid and easy to administer, to be minimally invasive, and to ensure consistency in scoring across the post-stroke recovery period. To assess health status and sickness behavior, the animal was observed in its home cage for general appearance, posture, respiration, and spontaneous locomotion/social interaction. Body condition (emaciation and hydration) was assessed by the pinch test. Body temperature and body weight were also measured (see Additional file 1: Table S1 for scoring criteria). The screen was administered twice prior to tMCAO and each day post-tMCAO for 7 days between 0800 and 0930.

### Modified Neurological Severity Score (mNSS)

The mNSS, adapted from Chen et al. [5], assessed neurologic function on a scale of 0–12 with 0 being normal and 12 being maximal deficit (see Additional file 1: Table S2 for scoring criteria). The mNSS used here was composed of motor and balance tests; the reflex test was eliminated due to habituation of the mice after repeat testing. mNSS scores were determined twice prior to tMCAO and each day post-tMCAO for 7 days between 0800 and 0930.

### Cylinder test

The cylinder test evaluates spontaneous forelimb use and asymmetries resulting from neural injury [6]. The animal was placed in a transparent cylinder (12 cm wide, 19 cm tall) for 5 min and allowed to freely explore. Performance was video recorded for later assessment. The number of independent wall placements for the right and left forelimbs, in addition to placement of both forelimbs simultaneously, were recorded. Cylinder test performance was conducted twice prior to tMCAO to account for pre-operative handedness biases, as well as 1, 3, 5 and 7 days post-tMCAO.

### Open field

This task evaluates locomotor activity and emotional reactivity [7]. A white plastic box (60 cm × 60 cm × 15 cm) was placed upon a table. Five desk lamps (60 watt each) provided indirect illumination. At the start of the trial, each mouse was placed in arena at the midpoint of the South wall and received a 6 min session. Locomotor activity was recorded using Ethovision 8.5 (Noldus Information Technology, Wageningenm, The Netherlands). For analysis, the box floor was divided into inner zone, middle frame, and perimeter zones. The dependent variables were fecal boli excreted, and distance moved (cm) in the arena as well as distance moved (cm) in each zone.

### Elevated plus maze

This task assesses anxiety-like behavior in a novel environment, with mice spending less time in open arms considered ‘anxious’ [8]. Two intersecting arm pairs were arranged in a ‘plus’ configuration and elevated 60 cm from the floor. One arm pair, 58 cm long × 5 cm wide, was enclosed by walls 15 cm tall and the other arm pair remained open (no walls or edges). Room illumination consisted of ambient lighting. Each mouse was placed at the arm intersection point and allowed to freely explore for 5 min. The dependent variables were the frequency of entries into each arm pair, the time spent (s) in the open arm pair, and the number of fecal boli excreted.

### Visible platform

This task confirms visual and motor competence for water maze testing [9]. A circular tub (140 cm in diameter) was filled with clear water. A white- and black-colored platform with a white/black flag was positioned approximately 0.5 cm above the water surface in a fixed location within the pool. Blue curtains covered obvious extra maze cues. The drop-off location varied semi-randomly across trials. Each mouse had 90 s to locate the platform, where it remained for 15 s before being placed back into its heated cage. If an animal did not locate the platform within the allotted time limit, it was gently guided to the platform. Between animals, the maze was cleaned of debris and olfactory cues were disrupted using a fishnet. The inter-trial interval was approximately 12 min. Latency (s) to reach the platform was the dependent measure.

### Morris water maze

*Acquisition* The Morris water maze tests hippocampal-dependent spatial reference memory [10], memory for information that remains consistent across time [11]. A round tub (140 cm in diameter) was filled with room temperature water made opaque with non-toxic paint. Spatial cues (shelving, door, striped posters, etc.) were indirectly lit. Briefly, the mouse was placed in the maze from any of four locations (North, South, East, or West) and had 60 s to locate a hidden platform submerged 1 cm, which remained in a fixed location (Northwest quadrant; NW). If an animal failed to locate the platform, it was gently guided to the platform. After 15 s on the platform, the mouse was placed into its heated cage until the next trial. Between animals, the maze was cleaned of debris and olfactory cues. There was approximately a 15 min inter-trial interval between trials. A tracking system (Ethovision XT 8.5) analyzed each mouse’s swim path. Animals received 4 trials for 6 days. The dependent measure was swim distance (cm). Swim speed (cm/s) was assessed to determine possible locomotor differences.

*Probe Trial* To assess platform localization, on the 6th day of testing an additional probe trial was given (trial 5), whereby the platform was removed from the maze. Percent of total swim distance (cm) in the target NW quadrant (i.e., quadrant that once contained the platform) versus the opposite Southeast (SE) quadrant and frequency of platform zone crossings were the dependent measure [12].

*Reversal* To assess cognitive flexibility/perseveration [13], reversal learning was assessed the following day, whereby the platform was relocated to the SE quadrant. Animals received 4 trials for 2 days, with a 15 min delay between trials three and four. Trial 5 was a probe trial on day 2. All testing procedures were identical to the Morris water maze acquisition and probe testing.

### Accelerating rotarod

This task assesses locomotor performance and coordination. A textured plastic horizontal rod (3 cm in diameter) was mounted 14.5 cm above a pressure-sensitive base (Ugo Basile). Acceleration was set to 4–44 rpm in 300 s [14]. For a given trial, a mouse was placed on the rod and the motor and timer switch were activated. Acceleration continued until the mouse fell onto the padded base or until 300 s had elapsed. Animals received 4 trials per session, with an inter-trial interval of 20 min, and two sessions per day, with an inter-session interval of 1 h, for 4 days. Latency to fall (s) was the dependent variable.

### Modified porsolt forced swim test

This test assesses depressive-like behavioral despair [15]. A plastic cylinder (19 cm tall, 12 cm wide) was filled up to a height of 10.5 cm with 25 °C water. Each animal was tested for 6 min. Following each trial, the animal was removed, dried with paper towels, and warmed in cages heated with a heat pad. The water was changed after each trial. Behavior was video recorded and quantified by a blind observer who scored each animal as either engaging in (1) active escape behaviors (swimming, climbing, and diving) or (2) immobility (floating or minimally moving). Behavior of each animal during the first 2 min was not included. Latency (s) to first immobility, total time (s) spent immobile, and number of fecal boli excreted were the dependent variables.

### Statistical analysis

Statistical analyses were performed with Statview. Differences between groups were analyzed by the unpaired Student’s *t* test, two-way ANOVA or a repeated measure two-way ANOVA as appropriate for each outcome measure (indicated in the figure legends).

## Results

### Infarct area

**Fig. 2:**
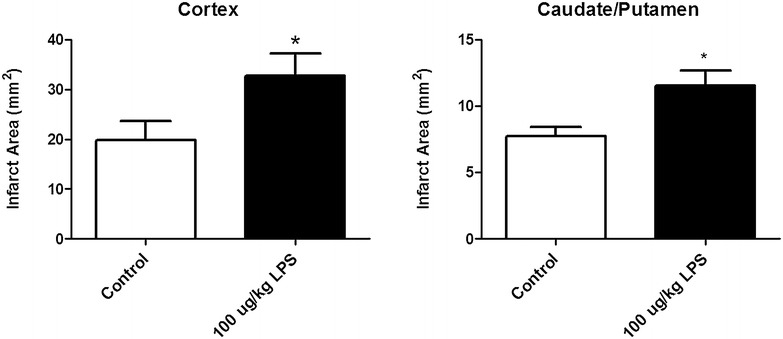
LPS exacerbates infarct area. *Cortical* and *Caudate/Putamen* infarct area. A student’s t-test between control and treatment group showed a significant increase in cortical infarct area (p = 0.0488) and caudate/putamen infarct area (p = 0.0134). Depicted are mean ± SEM for 7 mice/group. *p < 0.05

Low dose bacterial infection mimic administered 30 min prior to a mild (30 min) tMCAO increased cortical infarct area (p < 0.05) and caudate/putamen infarct area (p < 0.05) (Fig. 2). We measured rectal temperature at 0, 6, 12, 18, 24, and 48 h post-stroke to ensure that the increase in infarct volume was not due to an increase in temperature after stroke, and this data was reported in Doll et al. [4] Stroke alone caused a modest decline in rectal temperature, while LPS + stroke caused a marked hypothermia post-stroke [4]. These data indicate that the observed increase in infarct volume caused by the combination of LPS and tMCAO was not due to a febrile response. As such, we then conducted assessments in an additional cohort of mice for the effects of LPS on the behavioral outcomes of stroke.

### Modified Neurological Severity Score

**Fig. 3:**
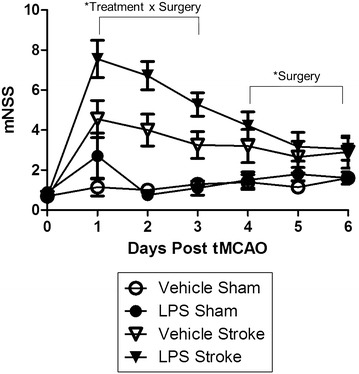
Stroke and Stroke + LPS exacerbate neurological deficits. A two way repeated measures ANOVA showed a significant day by treatment (p = 0.0067) and day by surgery effect (p < 0.0001). When blocking the data, day 1–3 (acute phase recovery) and day 4–6 (sub-acute phase recovery), there was a significant treatment by surgery interaction (0.0454) during the acute phase of recovery. This significant treatment by surgery interaction was lost during the sub-acute phase of recovery. Depicted are mean ± SEM for 9–10 mice/group. When SEM is not shown, it is smaller than the symbol used to depict the mean. *p < 0.05

To assess functional neurological damage, the modified neurological severity score (mNSS) adapted from Chen et al. [5] was administered for 7 days post-surgery. Prior to surgery, the mNSS was administered to assess baseline biases, and animals in all 4 groups scored similarly (p = 0.6652; F [1, 35] = 0.1904). Overall there was a day × treatment (p = 0.0067; F [1, 35] = 2.875) and day × surgery interaction (p < 0.0001; F [1, 35] = 13.603). Upon visual inspection of the graph, the stroke and LPS effect diminished over time resulting in the effect of surgery or treatment being exacerbated in the acute recovery phase (days 1–3) (Fig. 3), but in the sub-acute recovery phase (days 4–6), mNSS scores returned to baseline levels. This rapid recovery after an insult in mice has been noted previously [16, 17]. Thus, we blocked the data by acute recovery phase (days 1–3) and sub-acute recovery phase (days 4–6) to determine the effect of treatment, surgery, and their interaction on neurological deficits. During the first 3 days post-surgery, there was an overall treatment × surgery interaction (p = 0.0454; F [1, 35] = 4.304) indicating that LPS worsens neurological deficits post-stroke. However, during the sub-acute recovery phase the treatment × surgery interaction was not observed (p = 0.7531; F [1, 35] = 0.101), but the Surgery effect remained (p < 0.005; F [1, 35] = 11.6).

### Health and sickness behavior screen

**Fig. 4:**
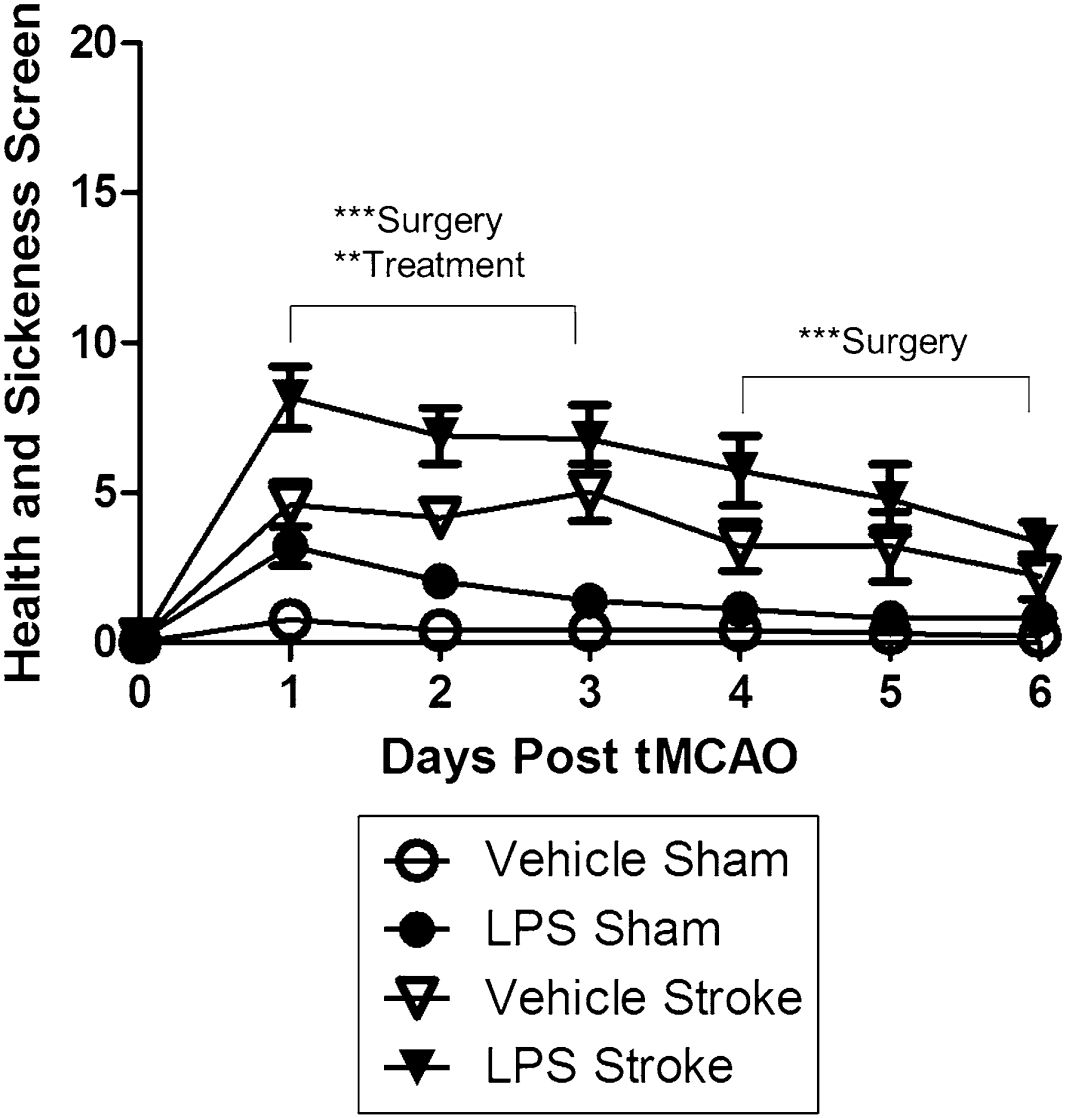
Stroke induces sickness behavior. A two way repeated measures ANOVA showed a significant day by treatment (p = 0.0006) and day by surgery effect (p < 0.0001). When blocking the data, day 1–3 (acute phase recovery) and day 4–6 (sub-acute phase recovery), there was a significant day by surgery effect (p < 0.0001). Depicted are mean ± SEM for 9–10 mice/group. When SEM is not shown, it is smaller than the symbol used to depict the mean. **p < 0.01; ***p < 0.001

Prior to tMCAO (time point 0), the animals in all four groups had a health screen score of 0 indicating that all animals were healthy animals at the time of treatment administration and/or stroke surgery (Fig. 4). Overall, there was a day × treatment (p < 0.005; F [1, 35] = 4.112) and a day × surgery interaction (p < 0.0001; F [1, 35] = 6.552). Upon visual inspection of the graph, the surgery and treatment effects were diminished over time, similarly to the mNSS. Thus, we blocked the data by acute recovery phase (days 1–3) and sub-acute recovery phase (days 4–6) to determine the effect of treatment, surgery, and the interaction on sickness behavior. Overall there were no interactions between stroke and LPS during the acute or sub-acute recovery phase. During both block of days there was a significant surgery effect (p < 0.0001; F [1, 35] = 63.974); however, only during the acute phase was there a significant treatment effect (p < 0.0005; F [1, 35] = 14.828). By the sub-acute recovery, the treatment effect diminished (p = 0.0842; F [1, 35] = 3.160), but the Surgery effect remained (p < 0.0001; F [1, 35] = 22.655).

### Motor function

**Fig. 5:**
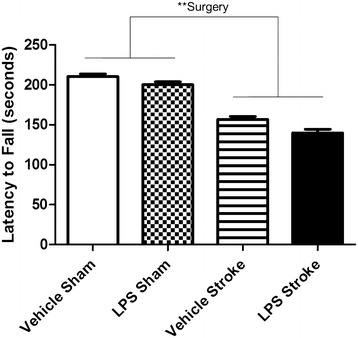
Stroke increases motor dysfunction. A two way repeated measures ANOVA showed a significant surgery effect (p = 0.0011). Depicted are mean ± SEM for 9–10 mice/group. **p < 0.01

**Fig. 6:**
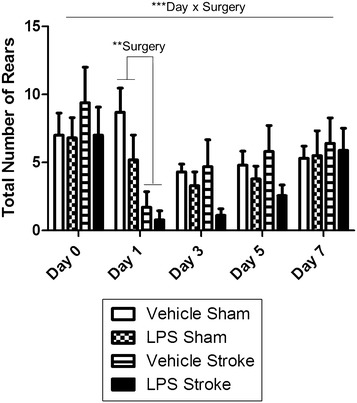
Stroke decreases rearing in the cylinder task. A two way repeated measures ANOVA showed a significant day by surgery effect (p < 0.0001). Further investigation of each day indicated a significant effect of surgery on day 1 (p = 0.0004) but by day 3 there was no effect of surgery or treatment. Depicted are mean ± SEM for 9–10 mice/group. **p < 0.01; ***p < 0.001

To assess motor function, performance on the accelerating rotarod was assessed 11 ± 1 days post-surgery. There was a significant stroke effect resulting in decreased motor function (p < 0.005; Fig. 5). This effect of stroke surgery on motor function was not due to decreased locomotor activity because open field indicated no differences between the groups (Table 1). To assess forelimb functional asymmetries, the cylinder task was administered. Before surgery (day 0), there were no differences between groups on rearing (Fig. 6) nor on left versus right forelimb placement (data not shown). There was an overall day × surgery effect (p < 0.0001; F [1, 35] = 8.916) post-surgery (Fig. 6); thus, we further investigated the effect of surgery on each day. There was a significant Surgery effect (p < 0.0005; F [1,35] = 15.23) on rearing on day 1, indicating that animals that had undergone stroke (regardless of drug treatment) were less active than animals who received sham surgery. By day 3, the surgery effect diminished. This finding of depressed locomotor behavior (as measured by overall number or rears) limits the interpretation of paw placement findings.

**Table 1:** Stroke or LPS does not have an effect on locomotor ability in open field

Group	Total distance (cm)	Center duration (s)	Inner duration (s)	Outer duration (s)
Vehicle sham	2295 ± 231	5 ± 2	15 ± 3	340 ± 5
LPS sham	2384 ± 208	5 ± 1	12 ± 3	343 ± 5
Vehicle stroke	2521 ± 257	5 ± 1	16 ± 5	339 ± 6
LPS stroke	2553 ± 232	6 ± 1	16 ± 2	338 ± 3

Reported are mean ± standard error of mean for total distance moved (cm), center duration (s), inner duration (s), and outer duration (s) during the open field task for each group

### Affective behaviors

Stroke nor LPS impacted general locomotor ability nor anxiety-like behavior in the open field (Table 1). There were no significant group differences in distance moved in any zone during the 6 min trial (Table 1). Similarly, stroke nor LPS impacted anxiety-like behavior on the elevated plus maze. There were no group differences in the number of open arm entries, number of closed arm entries, or the time (s) spent in the open arms (data not shown). Moreover, neither stroke nor LPS impacted depressive-like behavior on the forced swim test. There were no significant treatment effects on total time spent immobile, or latency to first immobility (data not shown).

## Discussion

Recent studies have indicated that both *Helicobacter pylori (H. pylori)* [18] and human cytomegalovirus (HCMV) [19] infections are associated with increased risk for ischemic stroke. Moreover, having a respiratory tract infection increases the risk for an atherothrombotic ischemic event for up to 3 months [20] and chronic bronchitis increases risk for stroke and transient ischemic attacks (TIA) [21]. Lastly, Grabska et al. [22] conducted a retrospective chart review of 2066 ischemic stroke patients to assess the effect of pre-stroke and post-stroke infection on ischemic stroke severity and found that pre-stroke infection increased poor outcomes within the first 30 days but did not have effects at 90 days on modified Rankin scores. From a preclinical perspective, LPS has been shown to increase infarct volume when administered prior to MCAO in rodents [3, 4]. However, the long-term effects of stroke during an active infection have not been studied previously. The present study shows for the first time that low dose LPS increases and prolongs sickness behavior and worsens neurological deficits post-stroke. Thus, infection appears to both trigger and worsen severity of stroke.

It is known that a severe stroke results in motor, cognitive, and affective behavioral deficits [16, 23–26]. Further, studies have shown that high doses of LPS causes motor, cognitive, and affective deficits [27–32]. However, until the current study, the long-term functional outcomes of stroke in combination with an active infection had not been methodically assessed in the rodent model. We hypothesized that the LPS administered prior to stroke would exacerbate the motor, cognitive, and affective behavioral deficits post-stroke. Our most robust effects of low dose LPS + mild stroke were on lesion volume, mNSS and sickness. The hypothermic response to LPS + stroke could be an adaptive protective response, because in other studies, we observed that maintenance of core body temperature following LPS + stroke resulted in profound sickness and death of mice (Doll et al., unpublished observations) and that acute post-stroke hypothermia is strongly associated with larger infarct size in mice (Heng et al., unpublished observations). The LPS + stroke effects on mNSS and sickness peaked at 1 day after stroke, then recovered over the 7-day observation period, indicating that acute behavioral response to LPS + stroke is transient, as has been previously reported for stroke alone in rodents and humans [6, 22]. We also showed that stroke, but not LPS treatment, affects balance and motor coordination during the accelerating rotarod task. As well, we did not observe effects of stroke, LPS, or their combination on open field, elevated plus maze or forced swim. Taken together, we demonstrate for the first time that an infection, present at the time of brain trauma (induced by MCAO) can exacerbate detrimental stroke outcomes, negatively impacting health and significantly prolonging recovery.

Limitations of the present study are several. We did not observe some of the previously reported behavioral deficits that have been found following MCAO or LPS among rodents. This is most likely due to methodological differences between our work and previous studies including (1) the timing of post-manipulation assessments, (2) the severity of the model of brain insult used here, and (3) the low dose of LPS used in our study. Indeed, it is well recognized that there are critical periods for detecting post-stroke deficits [6]. As well, many studies assessing functional outcomes following LPS assessed performance within hours of treatment administration. Thus, we may have not seen LPS + stroke effects in some behaviors due to the timing of assessment. Also, severity of the stroke insult may have influenced our ability to detect functional deficits. Our stroke was a 30 min tMCAO, which is considered a minor insult, and caused a small infarct (Fig. 2). The low dose of LPS used here, and thus the severity of the infection induced at the time of stroke also may explain why we did not observe substantial detrimental behavioral changes. Although others have shown that LPS results in motor, cognitive, and affective behavioral deficits, the doses used ranged from 0.3–10 mg/kg which are 3–100 times higher than the dose used in the present study. Additionally, a few studies injected LPS multiple times before assessing behavior [30]; thus, the single, low LPS dose given in this study likely resulted in a minor bacterial infection mimic, and the chosen battery of functional assessments may have not been sensitive enough to detect a mild impairment at the time points at which they were administered. Future studies should assess different doses of LPS and determine the time to assess different behavioral deficits. Furthermore, an increase prevalence of strokes occurs during flu season. Here, we utilized LPS, a bacterial infection mimic. However, it is not clear whether the exacerbation of post-stroke functional deficits by LPS found here extend to other types of infection. Thus, the assessment of the effects of a viral infection mimic prior to stroke on functional outcome is needed.

This study was not designed to assess the mechanism(s) of the interaction between LPS and stroke, but rather to describe the consequences of this interaction on lesion volume, neurological deficits, sickness and behavioral outcomes. There are several potential mechanisms by which LPS could exacerbate stroke outcome. LPS itself is neurotoxic [33], and increases levels of several neurotoxic cytokines, including TNFα [3]. We have shown that TNFα causes neuronal loss by inhibition of mitochondrial oxidative phosphorylation [34]. Additionally, LPS compromised the blood–brain barrier (BBB) [4]. As such, through either or both mechanisms, treatment with low doses of LPS may sensitize animals to the subsequent effects of a mild stroke and thereby worsen outcomes.

Thus, we can conclude from this study that a minor bacterial infection mimic prior to a minor stroke results in increased infarct area, increased sickness behavior and worse neurological deficits resulting in delayed functional recovery. Our observations of the association between infection, stroke, and worse sickness and neurological outcomes argues for the need to aggressively treat infection in people with risk factors for stroke and the need to understand the mechanism(s) of this association.

## Additional file

10.1186/s12993-015-0077-5 Overall animal health within seven physiological domains were determined using the health screen, a tool we designed to be administered easily and rapidly, to be minimally invasive, and to provide consistency of scoring between groups of animals and within individual animals across the post-stroke recovery period.
