# Supplementary material for: Lipopolysaccharide exacerbates infarct size and results in worsened post-stroke behavioral outcomes
Source: Behav Brain Funct. 2015 Oct 13;11:32. doi: 10.1186/s12993-015-0077-5 (PMC4604642; doi:10.1186/s12993-015-0077-5)
Supplement: Supplementary file 1 — 10.1186/s12993-015-0077-5 Overall animal health within seven physiological domains were determined using the health screen, a tool we designed to be administered easily and rapidly, to be minimally invasive, and to provide consistency of scoring between groups of animals and within individual animals across the post-stroke recovery period. [file 12993_2015_77_MOESM1_ESM.docx]

| **Parameter** | **Observation** | **Score** |
| --- | --- | --- |
| General Appearance | Normal  Groomed, healthy appearing fur, pink mucous membranes and ear lobes | 0 |
|  | Mild Abnormal  Mildly rough/scruffy/dull fur, slightly less well-groomed, light pink mucous membranes/ear lobes, minimal porforin staining, slightly squinted eyes | 1 |
|  | Moderate Abnormal  Rough/scruffy fur, piloerection, poor grooming, pale mucous membranes and ear lobes, squinted eyes | 2 |
|  | Severe Abnormal  Very rough fur, no evidence of grooming, white mucous membranes and ear lobes, substantial porforin staining, severely squinted or closed eyes | 3 |
| Posture | Normal | 0 |
|  | Slight Hunch  Spine slightly curved | 1 |
|  | Moderate Hunch  Spine curved, paws slightly under body | 2 |
|  | Severe Hunch  Spine dramatically curved, paws tucked under body, head angled downward | 3 |
| Body Condition | Normal | 0 |
|  | Thin  Slight segmentation of vertebrae, dorsal pelvic bones are more prominent, slight dehydration (skin pinch test response is slightly delayed) | 1 |
|  | Emaciated  Prominent vertebrae and skeletal bones that are readily palpable, dehydrated (skin pinch test results in skin remaining tented) | 2 |
| Respiration | Normal | 0 |
|  | Altered  Increased rate and/or effort | 1 |
|  | Abnormal/Distressed  Very increased rate or gasping/labored breathing, irregular | 2 |
| Body Temperature | Normal/No change | 0 |
|  | 1-4 degree C loss | 1 |
|  | 5-8 degree C loss | 2 |
|  | 9-12 degree C loss | 3 |
| Body Weight | 0-5% loss | 0 |
|  | 5.1-10% loss | 1 |
|  | 10.1-15% loss | 2 |
|  | 15.1-20% loss | 3 |
|  | > 20.1% loss | 4 |
| Spontaneous Locomotion/Social Interaction | Normal  Active and interacting with cage-mate(s) | 0 |
|  | Mild Abnormal  Still spontaneous activity and some peer interaction but reduced | 1 |
|  | Moderate Abnormal  Lethargic (may need probing via tapping on cage or cage tilt) and minimal peer interaction | 2 |
|  | Severe Abnormal  Immobile and no peer interaction | 3 |

Supplemental Table 1: Health and Sickness Behavior Screen.

Supplemental Table 2: Modified neurological stroke severity score (adapted from Chen et al., 2005)

Subtest Score

Walking Test 3

Normal 0

Inability to walk straight 1

Circling toward paretic side 2

Falling down toward paretic side 3

Beam Balance Test 6

Balances steadily or traverses beam to clamp 0

Grasps sides of beam ( 60 > x > 50 sec) 1

Hugs beam and/or 1 limb slips/falls down ( 50 > x > 40 sec) 2

Hugs beam, 2 limbs slip/fall down and/or spins on beam ( 40 > x > 30 sec) 3

Attempts to balance but very unsteady (30 > x > 20 sec) 4

Attempts to balance but clings to underside of beam (20 > x > 10 sec) 5

No attempts to balance and falls ( > 10 sec) 6

Inverted Test 3

Forelimb flexion or limb not moving to aid with balance 1

Hindlimb flexion or limb not moving to aid with balance 1

Head moved more than 10 degrees from vertical center or persistent spin 1

Total = 12

***NOTE for Beam Balance: an animal that balances steadily but falls due to traversing or exploring the beam should still score close to zero. Similarly, an animal that balances poorly but stays on for the whole trial should score worse (4-6 range).


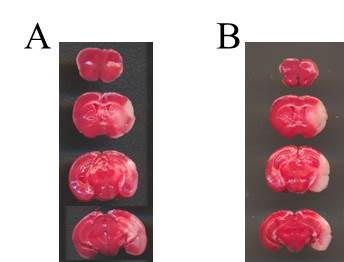


Supplemental Figure 1: Representative TTC stained brains. A) Saline administered 30 minutes prior a 30 minute tMCAo. B) 100 ug/kg LPS administered 30 minutes prior to a 30 minute tMCAo.
